# Influence of sportive activity on functional and radiographic outcomes following reverse total shoulder arthroplasty: a comparative study

**DOI:** 10.1007/s00402-022-04344-1

**Published:** 2022-01-29

**Authors:** Stephanie Geyer, Jakob Siebler, Felipe Eggers, Lukas N. Münch, Daniel P. Berthold, Andreas B. Imhoff, Sebastian Siebenlist, Bastian Scheiderer

**Affiliations:** Department of Orthopaedic Sports Medicine, Technical University of Munich, Ismaninger Str. 22, 81675 Munich, Germany

**Keywords:** Reverse total shoulder arthroplasty, Sports, Implant loosening, Scapular notching, Complications

## Abstract

**Background:**

The purpose of the present study was to compare the functional and radiographic outcomes following reverse total shoulder arthroplasty (RTSA) in a senior athletic and non-athletic population.

**Material and methods:**

In this retrospective cohort study, patients who underwent RTSA between 06/2013 and 04/2018 at a single institution were included. Minimum follow-up was 2 years. A standardized questionnaire was utilized for assessment of patients’ pre- and postoperative physical fitness and sportive activity. Patients who resumed at least one sport were assigned to the athletic group, while patients who ceased participating in sports were assigned to the non-athletic group. Postoperative clinical outcome measures included the Constant score (CS), American Shoulder and Elbow Surgeons (ASES) score, Simple Shoulder Test (SST), and visual analog scale (VAS) for pain. Active shoulder range of motion (ROM) and abduction strength were assessed. Radiographic evaluation was based on a standardized core set of parameters for radiographic monitoring of patients following shoulder arthroplasty.

**Results:**

Sixty-one of 71 patients (85.9%; mean age: 72.1 ± 6.6 years) were available for clinical and radiographic follow-up at a mean of 47.1 ± 18.1 months. Thirty-four patients (55.7%) were assigned to the athletic group and 27 patients (44.3%) to the non-athletic group. The athletic group demonstrated significantly better results for CS (*P* = 0.002), ASES score (*P* = 0.001), SST (*P* = 0.001), VAS (*P* = 0.022), active external rotation (*P* = 0.045) and abduction strength (*P* = 0.016) compared to the non-athletic group. The overall rate of return to sport was 78.0% at an average of 5.3 ± 3.6 months postoperatively. Incomplete radiolucent lines (RLL) around the humeral component were found significantly more frequently in the athletic group compared to the non-athletic group (*P* = 0.019), whereas the occurrence of complete RLLs around the implant components was similar (*P* = 0.382). Scapular notching was observed in 18 patients (52.9%) of the athletic group and 12 patients (44.9%) of the non-athletic group (*P* = 0.51). The overall rate for revision surgery was 8.2%, while postoperative complications were encountered in 3.3% of cases.

**Conclusion:**

At mid-term follow-up, the athletic population demonstrated significantly better clinical results following RTSA without a higher rate of implant loosening and scapular notching when compared to non-athletic patients. However, incomplete radiolucency around the humeral component was observed significantly more often in the athletic group.

**Level of evidence:**

III.

## Introduction

Historically, reverse total shoulder arthroplasty (RTSA) was designed for the treatment of pseudoparalysis with cuff tear arthropathy in elderly low-demand patients [2, 6, 17]. Over the past decades, indications have expanded comprising irreparable rotator cuff tears without osteoarthritis, primary osteoarthritis, acute proximal humerus fractures, fracture sequelae, and failed anatomic shoulder arthroplasty [5, 20, 22, 27]. Thus, younger and/or more active individuals have become more likely considered for RTSA [8]. These patients demonstrate higher expectations of shoulder surgery to participate in sports [18], and return to sport rates following RTSA have been reported to vary between 60 and 85% [15, 16, 25].

Yet, there remains a paucity of literature whether postoperative sportive activity, that places increased stress on the RTSA, leads to early implant loosening and mechanical complications. Simovitch et al. [25] reported good short-term clinical results following RTSA in a senior athletic population, without identifying prominent modes of mechanical failure in radiographic evaluation at a mean follow-up of 43 months. However, this study was limited to the lack of a non-athletic control group.

The purpose of the present study was to compare the functional and radiographic outcomes following RTSA in a senior athletic and non-athletic population. The authors hypothesized that athletic high-demand patients would demonstrate a higher rate of radiographic changes around the implant including radiolucency, loosening, and scapular notching when compared to non-athletic patients at a minimum two-year follow-up.

## Material and methods

### Study population

In this retrospective cohort study, patients who underwent RTSA using the Universe Reverse shoulder prosthesis (Arthrex Inc., Naples, FL, U.S.A.) between 06/2013 and 04/2018 at a single institution were included. Surgical inclusion criteria were cuff tear arthropathy, primary osteoarthritis, posttraumatic arthritis, and acute humeral head fracture. Minimum follow-up was 2 years. Patients were excluded for previous arthroplasty of the ipsilateral shoulder, vascular or malignant disease, and dementia.

A standardized questionnaire was utilized for assessment of patients’ pre- and postoperative physical fitness and sportive activity [16]. Patients who resumed at least one sport for more than two hours per week following RTSA were assigned to the athletic group, while patients who ceased participating in sports were assigned to the non-athletic group [16]. The local Institutional Review Board and the German Federal Office for Radiation Protection provided approval for the study (No. 269/19 s and No. Z5—22,464/2019–121-A).

**Fig. 1:**
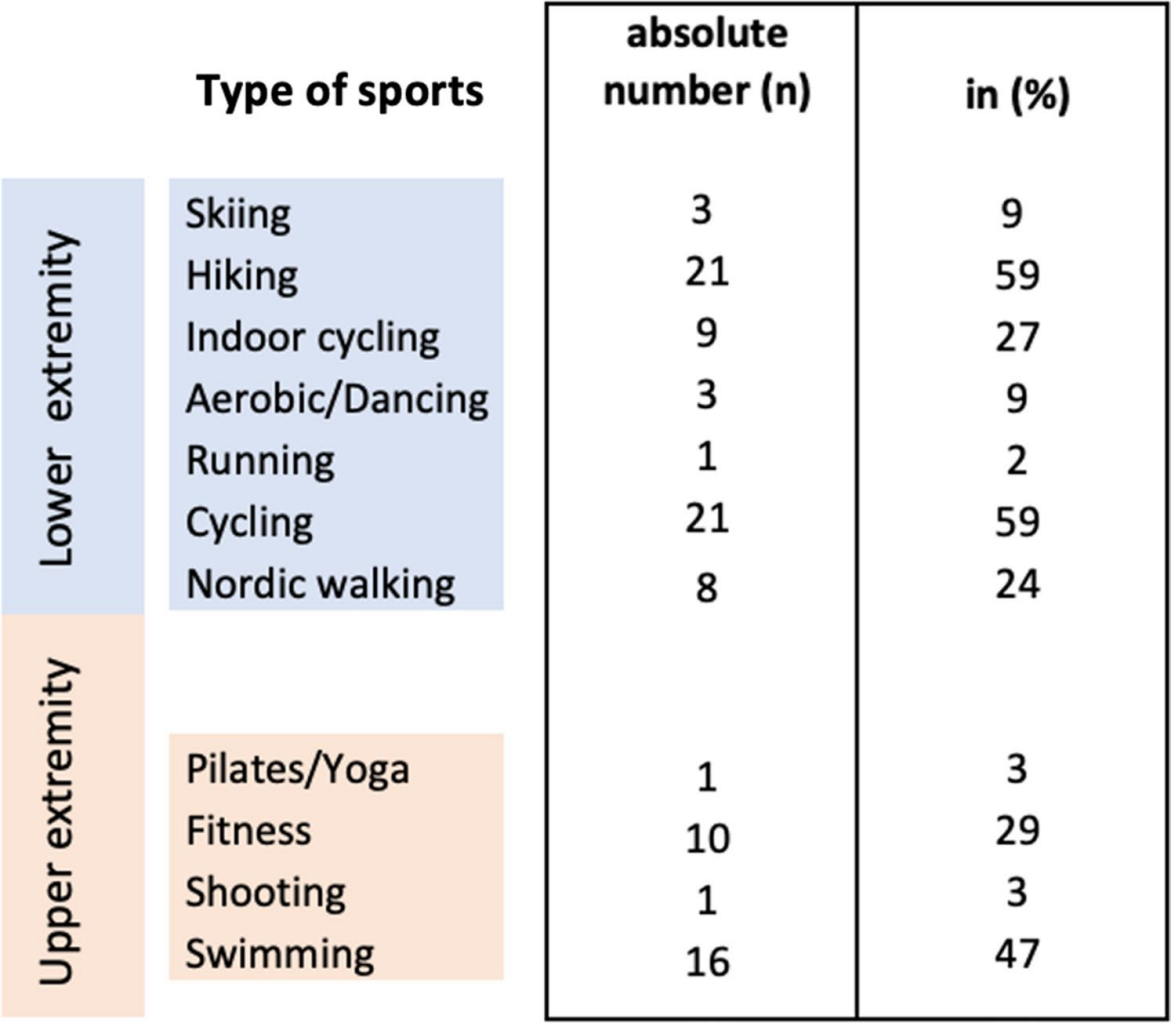
Sports performed in the athletic group following RTSA (multiple selection was possible)

### Surgical intervention and postoperative rehabilitation protocol

The deltopectoral approach was used in all patients. Glenosphere diameter was 36 mm in 12 patients (19.7%), 39 mm in 25 patients (41.0%), and 42 mm in 24 patients (39.3%). Glenosphere offset was standard (+ 0 mm lateral) in 48 patients (78.7%), and lateral (+ 4 mm lateral) in 8 patients (13.1%), whereas 5 patients (8.2%) had an inferior offset (+ 2.5 mm inferior). Structural bone-graft augmentation for severe glenoid bone loss was performed in 7 patients (11.5%). In 52 patients (85.2%) the humeral neck-shaft angle was 135°, and 155° in 9 patients (14.8%). All but 5 humeral stems (8.2%) were uncemented. In 44 patients (72.1%) the subscapularis tendon was repaired.

All patients followed a standardized rehabilitation protocol. Postoperative the shoulder was immobilized in an abduction brace for four weeks, along with active-assisted mobilization. Patients progressed with active range of motion and strengthening exercises at five weeks. A return to sports was permitted at four months postoperatively.

### Clinical outcome measures

Clinical outcome measures included the unadjusted as well as the age- and sex-adjusted Constant score (CS) [10, 11], the American Shoulder and Elbow Surgeons (ASES) score [23], the Simple Shoulder Test (SST) [19], and a 10-point visual analog scale (VAS) for pain [9], which were collected at a minimum follow-up of 2 years postoperatively. Shoulder active range of motion (ROM) was assessed using a goniometer with the patient in a standing position. Abduction strength was measured with an isometric dynamometer (Isobex, Cursor AG, Bern, Switzerland).

### Radiographic assessment

Radiographic examination included true anteroposterior, axial, and y-views for all patients, which were performed at each follow-up visit. Evaluation was based on a standardized core set of parameters for radiographic monitoring of patients following shoulder arthroplasty (Table 1) [12]. Radiographic grading was performed by two independent observers (SG, BS), the results were determined by consensus.

**Table 1:** Radiographic Parameters for Shoulder Arthroplasty Monitoring [12]

Parameters	Specifications and Definitions
Implant migration	*- Subsidence* None: no sign of subsidence Suspicion: subsidence is suspected but with no more than 5 mm of migration Definite: subsidence is noted with > 5 mm of migration *- Tilt* None: no sign of tilt Suspicion: tilt is suspected but with no more than 10 degrees of angulation Definite: tilt is noted with > 10 degrees of angulation *- Shift* Migration as a combination of subsidence and tilt. Shift is suspected when both subsidence and tilt are suspected or 1 is suspected and the other is definite. Shift is definite when both subsidence and tilt are definite
Radiolucency around the implant and implant loosening	Radiolucent lines (RLL) around the humeral and glenoid components. The humeral component is further divided into metaphysis and diaphysis Grade 0: none Grade 1: incomplete RLLs (radiolucency not all around the implant) a: no line reaching 1.5 mm in width b: at least 1 RLL reaching ≥ 1.5 mm in width Grade 2: complete radiolucency around the implant a: not reaching 1.5 mm in width b: reaching ≥ 1.5 mm in width (loosening)
Bone resorption and formation	*- Scapular notching*[26] Grade 1: defect limited to the scapular pillar Grade 2: defect in contact with the inferior screw of the base plate Grade 3: defect extending over the inferior screw of the base plate Grade 4: defect reaching the central peg of the base plate *- Heterotopic bone formation*[7] Grade 1: islands of bone within the soft tissue around the shoulder Grade 2: bone spurs from the proximal humerus or scapula, leaving at least 1 cm between opposing bone surfaces Grade 3: bone spurs from the proximal humerus or scapula, reducing the space between opposing bone surfaces to < 1 cm Grade 4: apparent osseous ankylosis of the shoulder

### Statistical analysis

Statistical means, minimum, maximum and standard deviations were calculated for continuous variables. Categorical variables were examined; frequencies and percentages were documented. The Mann–Whitney-*U* Test was used to compare the athletic group with the non-athletic group as a nonparametric test for shoulder scores (CS, ASES, SST and VAS), and radiolucent lines around the RTSA with a significance level of *P* < 0.05. The chi-square test or the fishers-exact test were used to compare radiographic changes including implant subsidence, tilt or shift, as well as scapular notching, and heterotopic ossifications with a significance level of *P* < 0.05. All statistical analysis was performed using SPSS Statistics, version 26 (IBM Corp., Armonk, N.Y., U.S.A.).

## Results

### Baseline characteristics

Sixty-one of 71 patients (85.9%) were available for clinical and radiographic follow-up at a mean of 47.1 ± 18.1 months. Mean age at the time of surgery was 72.1 ± 6.6 years. Thirty-six patients (59.0%) were females, and 25 patients (41.0%) were males. The dominant side was affected in 42 patients (68.9%). Twenty-seven patients (44.3%) had undergone prior surgery including rotator cuff repair (15 patients), open reduction and internal fixation (ORIF) of the proximal humerus or glenoid (10 patients), comprehensive arthroscopic management (CAM) procedure (1 patient), and shoulder resurfacing (1 patient).

Thirty-four patients (55.7%) were assigned to the athletic group and 27 patients (44.3%) to the non-athletic group. The groups were similar in terms of age, sex, body mass index (BMI), ASA (American Society of Anesthesiologists) score, side of surgery with respect to hand dominance, and follow-up. Baseline characteristics of the two groups are summarized in Table 2.

**Table 2:** Baseline characteristics

	Athletic Group (*n* = 34)	Non-athletic Group (*n* = 27)
Age^†^ (yr)	71.7 ± 5.4	72.5 ± 7.9
Sex (f/m)	19/15	17/10
BMI^†^	27.1 ± 4.0	29.4 ± 7.1
ASA score^†^	2.1 ± 0.8	2.3 ± 0.5
RTSA on the dominant side (no. [%])	23 (67.7)	19 (70.4)
Diagnosis leading to RTSA (%)		
Cuff tear arthropathy	52.6	54.5
Primary osteoarthritis	28.9	21.2
Posttraumatic arthritis	13.2	24.3
Acute humeral head fracture	5.3	0
Prior surgeries (%)	47.1	40.7
Follow-up^†^ (mo)	48.1 ± 18.9	45.8 ± 17.3

*BMI* body mass index, *ASA* American Society of Anesthesiologists, *RTSA* Reverse total shoulder arthroplasty

^†^Values are given as mean ± standard deviation

### Clinical outcomes

The athletic group demonstrated significantly better results for CS, ASES score, SST, VAS, active external rotation and abduction strength compared to the non-athletic group (Table 3).

**Table 3:** Postoperative outcome scores, range of motion, and strength measurement

	Athletic Group (*n* = 34)	Non-athletic Group (*n* = 27)	*P Value*
CS (points)	68.6 ± 10.4	55.2 ± 17.2	0.002
CSrel. (%)	96.4 ± 13.6	78.8 ± 25.8	0.002
ASES score (points)	87.0 ± 10.4	71.5 ± 21.3	0.001
SST	9.1 ± 2.0	6.8 ± 2.9	0.001
VAS	0.1 ± 0.3	1.3 ± 2.3	0.022
Range of Motion (deg)			
Active Flexion	134.7 ± 22.2	122.8 ± 28.5	0.166
Active Abduction	125.0 ± 22.3	116.2 ± 36.5	0.140
Active ER	29.6 ± 18.5	19.0 ± 17.6	0.045
Abduction strength (N)	3.7 ± 2.0	2.3 ± 1.7	0.016

*CS* Constant score, *CSrel* age and sex adjusted Constant score, *ASES* American Shoulder and Elbow Surgeons , *SST* Simple-Shoulder Test, *VAS* visual analog scale for pain, *ER* external rotation

Values are given as mean ± standard deviation

### Return to sport and sportive activities

Patients returned to sporting activities at an average of 5.3 ± 3.6 months postoperatively. Figure 1 summarizes the sports performed following RTSA. Postoperatively, among the 34 patients in the athletic group, 6 patients participated in sports every day, 20 patients ≥ 2 times a week, and 8 patients once a week. Eighteen patients (52.9%) indicated that they were able to perform at a higher level, 5 patients (14.7%) were only able to perform at a lower level, and 11 patients (32.4%) reported no change in ability compared with the status prior to undergoing RTSA. Thirteen patients (38.2%) reported residual pain in the operated shoulder while participating in sports with a mean VAS of 1.7 ± 1.0. Thirteen patients (38.2%) continued sports but gave up overhead sports including tennis, golf and climbing. The reasons for giving up those activities were fear in 21.1%, missing confidence in 31.6%, insufficient ROM in 21.1%, pain in 15.8%, and the surgeon’s recommendation in 10.5% of cases.

### Radiographic outcomes

Incomplete radiolucency (Grade 1a and 1b) around the humeral component metaphysis was observed in 16 patients (47.1%) of the athletic group and 4 patients (14.8%) of the non-athletic group (*P* = 0.008); and around the humeral component diaphysis in 12 patients (35.3%) of the athletic group and 2 patients (7.4%) of the non-athletic group (*P* = 0.01).

Complete radiolucency (Grade 2a and 2b) around the humeral component metaphysis was observed in 1 patient (2.9%) of the athletic group and 2 patients (7.4%) of the non-athletic group (*P* = 0.423). There were no complete radiolucent lines (RLLs) around the humeral component diaphysis in patients of the athletic group, but in 2 patients (7.4%) of the non-athletic group (*P* = 0.107). One patient (3.7%) of the non-athletic group demonstrated loosening (RLLs ≥ 1.5 mm around the humeral component metaphysis and diaphysis) and shift of the stem.

Incomplete radiolucency around the glenoid component was observed in 1 patient (2.9%) of the athletic group, and 3 patients (11.1%) of the non-athletic group (*P* = 0.2). There was no loosening and/or migration of the glenoid component.

Incomplete radiolucency around the humeral component was found significantly more often in the athletic group compared to the non-athletic group (*P* = 0.019), whereas the occurrence of complete RLLs around the implant components demonstrated no statistical difference between the two groups (*P* = 0.382).

Scapular notching was observed in 18 patients (52.9%) of the athletic group and 12 patients (44.9%) of the non-athletic group (*P* = 0.51). Eleven patients (61.1%) in the athletic group had grade 1 scapular notching, 6 patients had grade 2, and 1 patient grade 4. In the non-athletic group 6 patients demonstrated grade 1 scapular notching, and 6 patients grade 2.

**Fig. 2:**
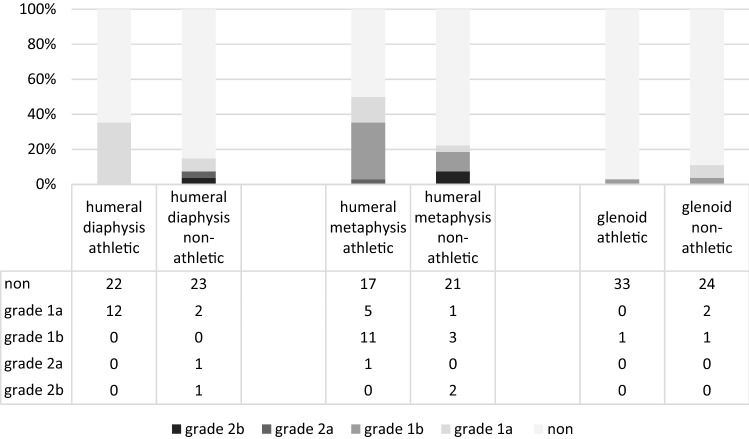
Radiolucency and implant loosening (grades are defined in Table 1)

**Fig. 3:**
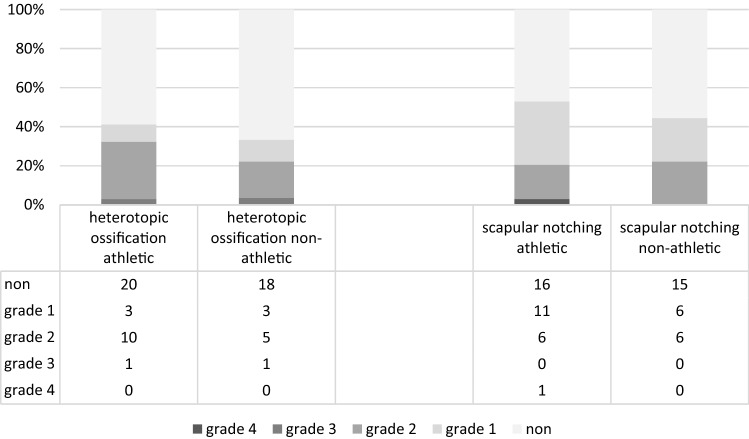
Bone resorption and formation: heterotopic ossification and scapular notching (grades are defined in Table 1)

Fourteen patients (41.2%) in the athletic group had heterotopic ossifications, and 9 patients (33.3%) in the non-athletic group (*P* = 0.53). Radiographic results are summarized in Figs. 2 and 3.

### Revisions and complications

A total of 5 patients (8.2%; athletic group *n* = 2; non-athletic group *n* = 3) had to undergo revision surgery, including 1 postoperative hematoma requiring soft-tissue revision, 1 iatrogenic periprosthetic humerus fracture treated with ORIF, and 1 traumatic dislocation requiring open reduction and exchange of the polyethylene liner. In two patients a two-stage revision arthroplasty was performed due to late infection.

Postoperative complications, other than the reported revision surgeries, were encountered in 2 patients (3.3%). One patient demonstrated asymptomatic loosening and migration of the humeral stem. One patient had an axillary nerve palsy that resolved spontaneously.

## Discussion

The most important finding of the present study was that the athletic population demonstrated significantly better clinical results following RTSA without a higher rate of implant loosening and scapular notching when compared to non-athletic patients at mid-term follow-up. However, incomplete radiolucency around the humeral component was observed significantly more frequent in the athletic group.

Simovitch et al.[25] reported clinical improvement following RTSA in a senior athletic population without radiographic decline or failure at a mean 43 months follow-up. As radiolucency around the humeral stem was observed to occur in only 17% of patients without development of stem or glenoid loosening along with a 7% rate of scapular notching, the authors concluded that it is relatively safe for a senior athlete to return to non-contact, as well as low- and high-impact sports. In the present study, the presence of any grade of radiolucency around the humeral stem (55.9%) was notably higher in the athletic population. However, comparison to our results is limited, as the study by Simovitch et al. [25] lacked of a classification for radiolucency.

 Endell et al. [13] compared the impact of sportive activity on radiographic outcomes for three RTSA patient groups (sports mainly involving upper extremity, sports mainly involving lower extremity and no sports at all). At a similar follow-up period the present study demonstrated significantly better clinical results in athletic patients following RTSA without a higher rate of radiographic implant loosening and scapular notching when compared to non-athletic patients at a mean follow-up of 47 months. Further, the athletic group demonstrated a significantly greater range of active external rotation and significantly higher strength in shoulder abduction. In previous work, the positive correlation of shoulder strength with sportive activity level and daily function has already been highlighted [28]. Endell et al. [13] also reported higher but not statistically relevant scores for patients performing sports mainly involving upper extremity without additional signs of implant using due to increased shoulder use.

As the eagerness and enthusiasm of athletic patients to return to their respective sportive activity potentially results in better compliance and motivation during postoperative rehabilitation, this may be an explanation for the significantly better functional outcomes in this group when compared to the non-athletic group. In the present study, 52.9% of patients indicated that they were able to perform sports at a higher level, while 14.7% of patients were only able to perform at a lower level compared with the status prior to RTSA. Garcia et al.[16] reported a similar development regarding intensity and duration of sportive activities following RTSA . Although residual pain in the operated shoulder while participating in sports was reported by 38.2% of patients, the mean VAS was only 1.7 ± 1.0. Interestingly, patients who performed overhead sports such as golf or tennis did not resume to those activities. The reasons were fear in 21.1%, missing confidence in 31.6%, insufficient range-of-motion in 21.1%, pain in 15.8%, and the surgeon’s recommendation in 10.5% of cases.

As variations in implant designs affect the location of the center of rotation along with overall joint mobility and stability [1], only patients who underwent surgery with one particular RTSA system were included in the study. Although this implant is widely used, clinical and radiographic outcomes are limited. Recently, a multicenter case series reported sufficient postoperative improvement in shoulder function and quality of life with the Universe Reverse shoulder prosthesis [24]. The overall complication rate was 25% with only 2.7% considered to be severe. Similarly, our data ﻿demonstrated﻿ an overall complication rate of 11.5% with a surgical revision rate of 8.2%. These findings are also consistent with general complication rates following RTSA, which have been shown to occur in 10 to 25% of cases [3, 4, 24].

In the present study the rate of scapular notching (49.2%) was higher compared to results reported by Schwyzer et al. (10.6%) [24]. This may be explained by their shorter minimum follow-up of 23 months, as Lévigne et al. [21] and Ernstbrunner et al. [14] described a progressiveness in frequency of scapular notching over a longer period of time. We observed similar rates of scapular notching when comparing the athletic and non-athletic group (52.9% and 44.9%, respectively). Endell et al. [13] showed a high scapular notching rate in the non-sportive group (51.0%) without reaching statistical difference when compared to sportive groups (upper extremity sports: 35% and lower extremity sports: 29%, respectively).

There were several limitations to the study. First, the mean follow-up period was 47 months, precluding the definite assessment of functional and radiographic changes which are usually more likely to occur at longer follow-up periods of 5 and 10 years. Second, the number of patients in the athletic and non-athletic group was limited, thus not allowing for a sports-specific evaluation of functional outcomes and radiographic changes. In addition, sportive activities showed a tendency to alpine related sports including hiking, biking, nordic walking, and skiing. In nordic walking and skiing the upper extremity is involved for pushing, balancing and climbing. However, these sports are neither high-impact nor typical overhead sports. Thus, the impact of overhead sports on potential radiographic changes around the implant remains limited.

## Conclusion

At mid-term follow-up, the athletic population demonstrated significantly better clinical results following RTSA without a higher rate of implant loosening and scapular notching when compared to non-athletic patients. However, incomplete radiolucency around the humeral component was observed significantly more often in the athletic group.
